# Tribological Performance of Diamond Films with Different Roughnesses of Silicon Nitride Substrates and Carbon Source Concentrations

**DOI:** 10.3390/membranes12030336

**Published:** 2022-03-18

**Authors:** Feng Lu, Tianwei Liu, Xu Bai, Yuhou Wu, He Wang, Guangyu Yan

**Affiliations:** 1 School of Mechanical Engineering, Shenyang Jianzhu University, Shenyang 110168, China; lufeng72@126.com (F.L.); liutianwei97@163.com (T.L.); wuyh@sjzu.edu.cn (Y.W.); wanghe9095@163.com (H.W.); ygy0813@sjzu.edu.cn (G.Y.); 2 Joint International Research Laboratory of Modern Construction Engineering Equipment and Technology, Shenyang Jianzhu University, Shenyang 110168, China

**Keywords:** HFCVD, diamond films, Si_3_N_4_ substrates, roughness, carbon source concentrations, friction and wear

## Abstract

Diamond films were deposited on silicon nitride (Si_3_N_4_) substrates with three different roughnesses using the method of hot-filament chemical vapor deposition (HFCVD). The tribological properties of the film were studied by changing the deposition time, deposition distance, and methane (CH_4_) concentration. The friction coefficient, delamination threshold load, and wear rate of the diamond films were tested and calculated using the reciprocating friction and wear test under dry friction conditions. The results show that, when the deposition time is 12 h, the bonding force of the film is the lowest and the friction coefficient is the largest (0.175, 0.438, and 0.342); the deposition distance has little effect on the friction performance. The friction coefficients (0.064, 0.107, and 0.093) of nano-diamond films (NCD) prepared at a 40 sccm CH_4_ concentration are smaller than those of micro-diamond films (MCD) prepared at a 16 sccm CH_4_ concentration. The load thresholds before delamination of R_a_ 0.4 μm substrate diamond film are as high as 40 N and 80 N, whereas the diamond films deposited on R_a_ 0.03 μm substrates have lower wear rates (4.68 × 10^−4^ mm^3^/mN, 5.34 × 10^−4^ mm^3^/mN) and low friction coefficients (0.119, 0.074, 0.175, and 0.064). Within a certain load range, the deposition of a diamond film on a R_a_ 0.03 μm Si_3_N_4_ substrate significantly reduces the friction coefficient and improves wear resistance. Diamond film can improve the friction performance of a workpiece and prolong its service life.

## 1. Introduction

In recent years, the research of inorganic membrane has attracted people’s attention. Inorganic membrane has excellent properties such as robustness, physicochemical stability, and long-term performance. Jianzhou Du et al. [1] prepared porous alumina ceramic membranes on air bearings. The results showed that the load capacity increased from 94 N to 523 N when the porosity increased from 5% to 25%. The porosity is an important factor for improving the performance of an air bearing, and it can be optimized to enhance the bearing’s stability and load capacity. The tribological properties of diamond film are some of the key factors affecting the film properties. Diamond film is widely used in industrial production because of its superior properties.

Si_3_N_4_ ceramics are some of the strongest ceramic materials and have excellent thermal, mechanical, and chemical properties, as well as good wear resistance. These properties are essential for mechanical applications with demanding tribological properties. Si_3_N_4_ is commonly used in industrial applications such as plain bearings, cutting tools, and mechanical seals [2]. However, during the process of wear, the free surface of Si_3_N_4_ reacts with the surrounding moist air and converts to hydrated silica produced on the worn surface. This performance destroys the stability of Si_3_N_4_, and severe wear has been observed for the self-mated Si_3_N_4_ tribopair. Special measures for depositing thin films on Si_3_N_4_ reduce wear. Diamond films have excellent mechanical and tribological properties, such as high hardness, good wear resistance, and a low coefficient of friction, and have received extensive attention in mechanical and tribological, high-performance engineering surfaces [1,3]. Si_3_N_4_ ceramic is considered an ideal material for the deposition of diamond films (~1 × 10^−6^ K^−1^) due to its low thermal expansion coefficient (~2.9 × 10^−6^ K^−1^), which ensures the required bonding strength between diamond films and substrates [4,5,6,7]. The tribological properties of diamond films play an important role in the performance and service life of diamond-coated components [8]. HFCVD is one of the methods most commonly used to prepare diamond films, which is simple to operate and has a low cost and can realize a large area of deposition and the deposition of diamond films on complex, geometric surfaces.

Many researchers have studied the tribological properties of various diamond films. Abreu et al. [9] studied the friction properties of NCD deposited on Si_3_N_4_ substrates under different surface treatment states. The results showed that the threshold load before film delamination depends on the initial roughness of the substrate, and the NCD showed a slight wear state suitable for self-pairing, dry sliding conditions. Bin Shen et al. [8] deposited MCD and NCD on a Si_3_N_4_ substrate and conducted friction tests with different counterbodies. The results showed that the deposition of a diamond film on the Si_3_N_4_ substrate significantly reduces the friction coefficient, reduces the wear rate, and improves the wear resistance. Naichao Chen et al. [3] deposited a single-layer MCD, a single-layer sub-micron diamond film, and a multilayer MCD on the surface of Si_3_N_4_ and conducted friction tests. The results showed that the multilayer MCD had the highest wear resistance. However, research on the deposition of diamond films on substrates with different roughnesses is scarce. The tribological properties and mechanical application of diamond films under different substrate surface conditions are different. Therefore, it is beneficial to the wide application of diamond films in the engineering field to clarify the tribological properties of diamond films under different surface conditions.

MCD and NCD were deposited on Si_3_N_4_ substrates with different roughnesses using HFCVD. Diamond films with different structures were obtained by adjusting the deposition parameters, and the composition and surface morphology of the films were characterized. The tribological properties of diamond films with different roughnesses were studied by sliding tests under dry friction conditions.

## 2. Materials and Methods

Diamond films were deposited on a Si_3_N_4_ ceramic substrate (25 × 25 × 3 mm) with HFCVD technology using CH_4_ and hydrogen (H_2_) mixture as reaction gas. The Si_3_N_4_ substrates with three different roughnesses (0.03, 0.1, and 0.4 µm) were manufactured by the manufacturer. Before the test, roughness tester was used to measure the roughness of the substrate. We ensured that it conformed to test standards. Before preparation, the surface of the substrate needed to be pretreated. The substrates were degreased and cleaned in ultrasonic baths with acetone and ethanol, rinsed in deionized water, and dried in hot air. After cleaning, the substrates were exposed to diamond suspension (5 nm) for 30 min with ultrasonic to increase the nucleation sites for diamond film growth. The hot filaments consisted of seven tantalum wires 50 cm long, which were straightened and fixed by a high-temperature spring. The Si_3_N_4_ was placed below the hot filaments on the sample holder, which remained fixed. The tantalum wire was carbonized for 2 h before deposition. The gas pressure was set to 3 kPa, and the temperatures of the hot filaments and substrate were 2450 ± 50 °C and 850 ± 30 °C, respectively. The specific diamond film deposition parameters are shown in Table 1.

Hitachi S-4800 scanning electron microscope (SEM) was used to observe the surface morphology of diamond film. The fracture cross-section morphology of the sample was observed using a Zeiss Sigma 500 SEM. Shimadzu XRD-7000 X-ray diffractometer (XRD) was used to analyze the grain orientation of diamond film. The composition of the film was analyzed by Thermo Fisher DXR Microscope Raman spectrometer. The roughness of diamond film was analyzed by Taylor Hobson roughness tester. The friction and wear properties of the prepared diamond films were tested on a Rtec MFT-5000 tribotester, and Si_3_N_4_ ceramic balls (diameter 6.35 mm) were used as counterbody ball. The dry friction experiment was carried out at room temperature. During the friction and wear test, the reciprocating frequency was kept at 3 Hz, and the reciprocating friction stroke was 10 mm, which provided an average sliding speed of 0.06 m/s. The tribotests were performed on bare substrate and substrates coated with diamond films. The adhesion ability of diamond film was judged by analyzing the load threshold side of diamond film shedding. A 20 N normal load was applied to all diamond films for friction tests. After the test, wear rate was measured by Rtec UP-Sigma white light interferometer, and the wear tracks were observed by Keyence VHX-1000 ultra-depth-of-field optical microscope.

## 3. Results and Discussion

### 3.1. Properties of Diamond Films

#### 3.1.1. Surface Morphology of Diamond Films

**Figure 1:**
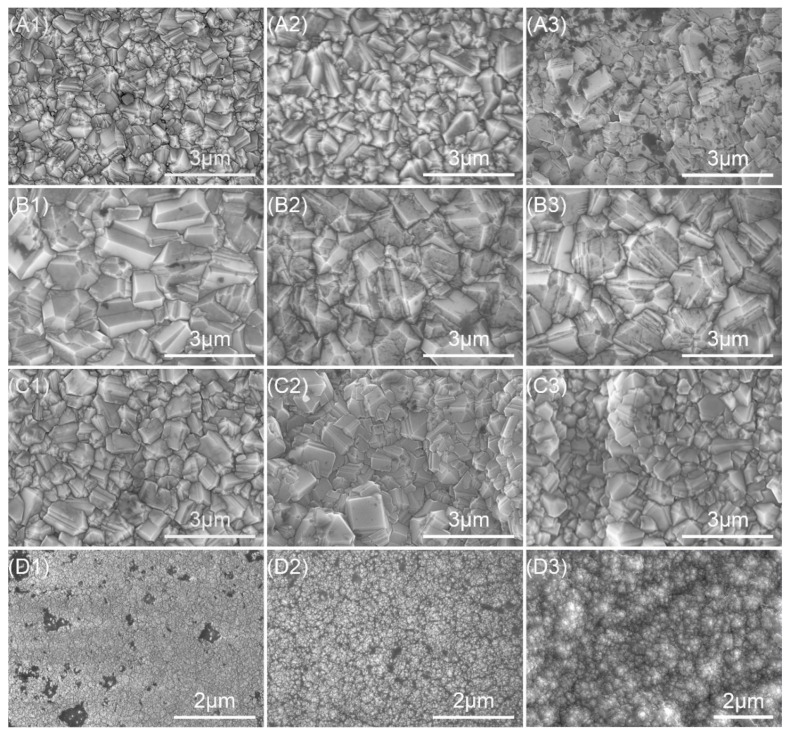
The surface morphology of diamond films.

Figure 1 shows the surface morphology of the diamond films with different deposition parameters. In Figure 1, A1 is sample A1 (R_a_ 0.03 μm, 16 sccm, 8 h, 4.5 mm), A2 is sample A2 (R_a_ 0.1 μm, 16 sccm, 8 h, 4.5 mm), A3 is sample A3 (R_a_ 0.4 μm, 16 sccm, 8 h, 4.5 mm), B1 is sample B1 (R_a_ 0.03 μm, 16 sccm, 8 h, 3 mm), B2 is sample B2 (R_a_ 0.1 μm, 16 sccm, 8 h, 3 mm), B3 is sample B3 (R_a_ 0.4 μm, 16 sccm, 8 h, 3 mm), C1 is sample C1 (R_a_ 0.03 μm, 16 sccm, 12 h, 4.5 mm), C2 is sample C2 (R_a_ 0.1 μm, 16 sccm, 12 h, 4.5 mm), C3 is sample C3 (R_a_ 0.4 μm, 16 sccm, 12 h, 4.5 mm), D1 is sample D1 (R_a_ 0.03 μm, 40 sccm, 5 h, 4.5 mm), D2 is sample D2 (R_a_ 0.1 μm, 40 sccm, 5 h, 4.5 mm), and D3 is sample D3 (R_a_ 0.4 μm, 40 sccm, 5 h, 4.5 mm). The following numbers have the same meaning as explained above. Sets A–C were MCD, and set D was NCD. When the CH_4_ concentration was 16 sccm, as shown in sets A–C, the microstructure of the diamond film was dense. Most of the grains in the sample had a triangular plane (111) as the outer surface, while a few A grains had a square plane (100), and a few A planes (100) were parallel to the sample plane, and the grains were angular. When the CH_4_ concentration was 40 sccm, as shown in the D set, with the increase in CH_4_ concentration, the grains in the film were refined, a large number of nano-grains were generated, and the plane of the film was optimized. The increase in CH_4_ flow causes the micro-grains to change into nano-grains, because the increase in CH_4_ flow reduces the etching ability of hydrogen ions and generates sp^2^ carbon at grain boundaries, which hinders the growth of diamond grains. As shown in set A, the deposition time was 8 h. The deposition time of the C set was 12 h. The grain size of both was about 1 μm, indicating that deposition time has little effect on the grain size. As shown in set A, the deposition distance was 4.5 mm, and the deposition distance of set B was 3 mm. The grain size of sample B was larger: about 2 μm. As shown in C1–C3, the roughness of the Si_3_N_4_ substrate was 0.03 μm, 0.1 μm, and 0.4 μm, respectively. It can be seen from the figure that the surface of the diamond film was also roughened sequentially after deposition.

**Figure 2:**
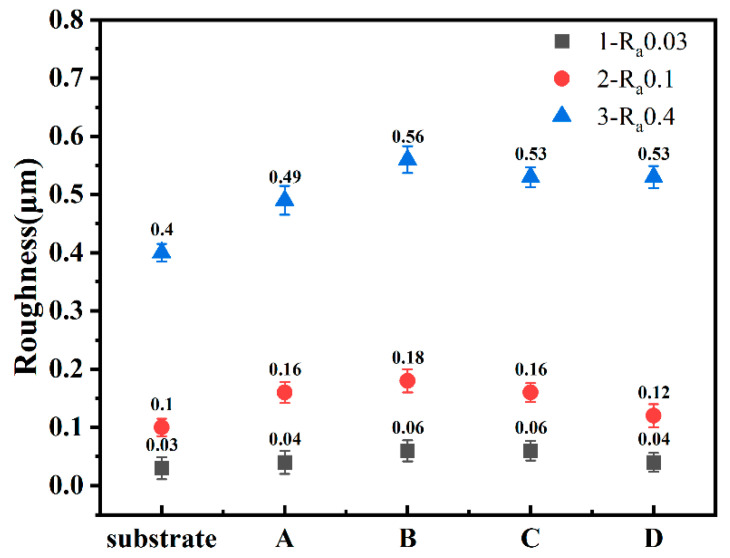
Roughness of diamond films.

Roughness is one of the important factors affecting the tribological properties of diamond film. Figure 2 shows the roughness of the diamond films prepared. The Si_3_N_4_ substrate used in the experiment was divided into three sets, and its roughness was 0.03 μm, 0.1 μm, and 0.4 μm, respectively. It can be seen from sets A–D that the roughness of the diamond film was similar to that of the Si_3_N_4_ substrate, and both of them were improved, which may be due to the fact that diamond grains grow in different directions on rough substrates, and the roughness is amplified by the size of diamond grains themselves [10]. The roughness of the diamond film is proportional to the roughness of the substrate. In sets 1 and 2, the roughness of the samples in set D was the lowest. This is because the roughness of the substrate is related to the size of diamond grains. The samples in set D were NCD with very small diamond grains, and the substrate was relatively smooth, which made the roughness smaller. From Figure 1 (D1,D2) it can be seen that the surface was relatively smooth; the roughness of the three set substrates was 0.4 μm, which is relatively rough, and the roughness of the prepared diamond film was mainly related to the roughness of the substrates [11], which were all around 0.5 μm. For sets A–C, the roughness of the diamond film was obtained by changing the deposition time and distance. It can be seen that the roughness of set B of samples was the largest. The set B test shortened the distance between the hot filament and the substrate. As can be seen from Figure 1, set B could also be used. It can be seen that the diamond grain size was the largest, indicating that the roughness has a certain relationship with the grain size. Through the measurement of roughness, it can be proved that the roughness of the substrate is one of the main factors of the roughness of the diamond film.

#### 3.1.2. Fracture Cross-Section Morphology of Diamond Films

**Figure 3:**
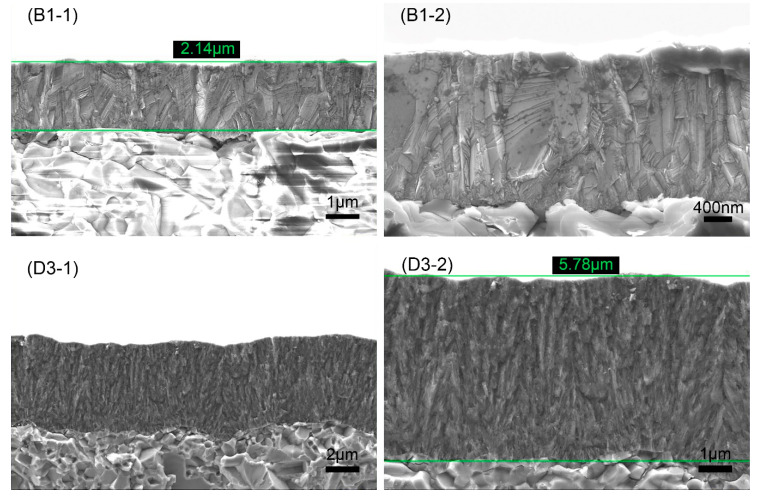
The cross-section morphology of MCD and NCD: (**B1-1**) the magnification of B1 sample was 10,000 times; (**B1-2**) the magnification of B1 sample was 20,000 times; (**D3-1**) the magnification of D3 sample was 5000 times; (**D3-2**) the magnification of D3 sample was 10,000 times.

Figure 3 shows the fracture cross-section morphology of B1 and D3 samples, where B1 is MCD and D3 is NCD. MCD grew in columnar form and coarsely grew into 1~2 μm grains from the finer grains at the bottom, while NCD grew in nanoclusters. The diamond grains were very small, and their obvious crystal shape could not be distinguished. The average growth rate of B1 and D3 was 0.27 μm/h and 1.16 μm/h, respectively. The growth rate of the diamond film increased with the increase in CH_4_ concentration, mainly due to the increase in methyl radical ([CH_3_]), which is considered to be the main source of diamond growth [12]. The deposition rate could be improved by increasing the CH_4_ concentration, and the quality of the diamond film could be reduced.

#### 3.1.3. Raman Spectra of Diamond Films

**Figure 4:**
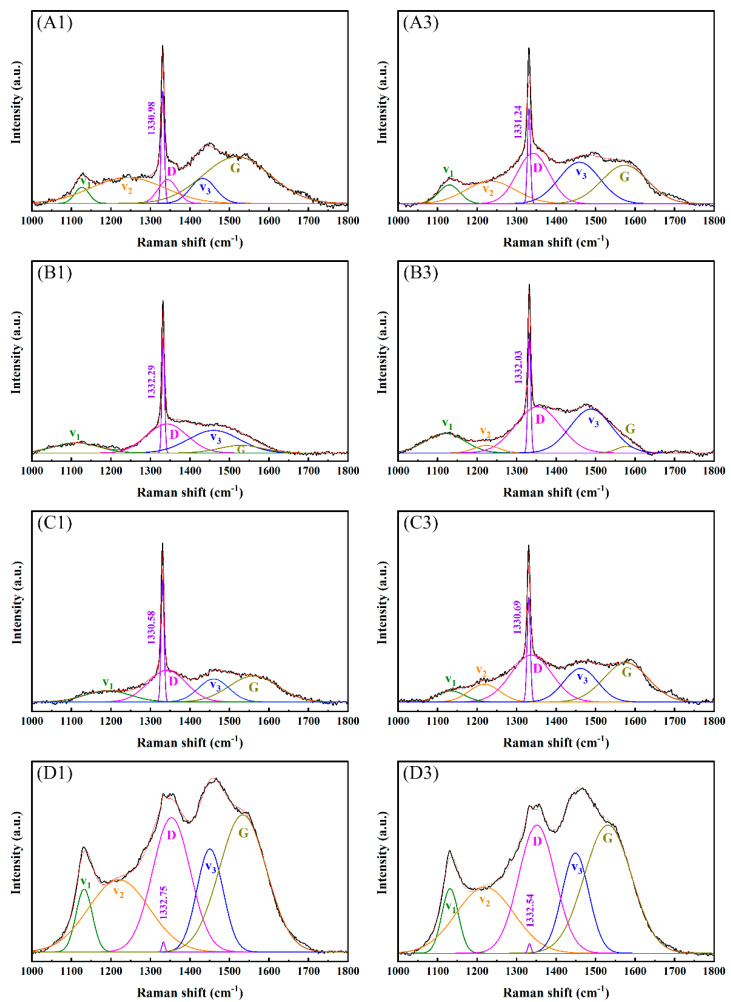
Raman spectra of diamond films deposited at different deposition parameters.

The purity and bonding method of the diamond film was judged according to the Raman peak intensity in the Raman spectrum. Figure 4 shows the Raman spectrum and fitting peaks of the diamond obtained under different deposition parameters. Raman peaks *v*_1_ (1150 cm^−1^), *v*_2_ (1250 cm^−1^), and *v*_3_ (1450 cm^−1^) indicate that there were nanodiamonds or trans-polyacetylenes in the diamond film [13]; D peaks (1350 cm^−1^~1360 cm^−1^) and G peaks (1550 cm^−1^~1580 cm^−1^) indicate the existence of an sp^2^ structure [14]; 1332 cm^−1^ is the characteristic peak of diamonds. When the concentration of CH_4_ was 16 sccm, as shown in Figure 4 for sets A–C of samples, the diamond’s characteristic peak intensity was large, and the surface film was mainly bonded by sp^3^ bonds. Combining with Figure 1’s sample sets A–C, it can be concluded that the diamond crystal quality is better. Among them, the trans-polyacetylene peak, D peak, and G peak are relatively weak, which indicates that the sp^2^ bonding content is low, and the grain boundary density of the diamond film is low.

When the CH_4_ concentration was 40 sccm, as shown in Figure 4 for sample set D, the characteristic peaks of diamond became weaker, mainly the D peak and the G peak, and the trans-polyacetylene peak was enhanced, indicating that the grain boundary density increased and the grain size decreased [15,16,17], and, as shown in Figure 1D’s set of samples, a large number of nanocrystalline diamonds were generated. This is because the increase in the CH_4_ concentration reduces the rate of hydrogen ion etching of graphite, resulting in an increase in the content of non-diamond phases, an increase in the probability of nucleation on the surface of the film, and the crystallization of the film. The quality decreases, and the intensity of the diamond’s characteristic peaks weakens. The Raman spectrum of sample sets A–C was similar, and it was very different from sample set D. In sample sets A–C, the deposition time and the distance from the hot filament to the substrate were adjusted, which had little effect on the film quality. Sample set D changed the CH_4_ concentration and affected the film quality. The influence was great, and it can be seen from Figure 1 that the surface morphology of the two was very different.

Equation (1) [18] calculates the residual stress in the film:

(1) $$\sigma =-0.567\;\left(v-v_{0}\right)\;\mathrm{GPa}/{\mathrm{cm}}^{-1}$$

where *σ* represents residual stress, and *v*_0_ represents the characteristic peak value of diamond at 1332 cm^−1^. *v* represents the diamond Raman displacement of the film. The right deviation of v over *v*_0_ is compressive stress, and the left deviation is tensile stress. Table 2 shows the results of the Raman spectroscopy analysis. The residual stress of 1 (R_a_ 0.03 μm) in sample set A–D was larger than 3 (R_a_ 0.4 μm) in the same set. The minimum residual stress of the B3 sample (R_a_ 0.4 μm, 16 sccm, 8 h, 3 mm) was −0.02 GPa, and the maximum residual stress of the C1 sample (R_a_ 0.03 μm, 16 sccm, 12 h, 4.5 mm) was +0.80 GPa, indicating that the deposition time increased. The residual stress also increased. The diamond characteristic peak, full-width at half-maximum (FWHM) of the continuous CVD diamond films synthesized on non-diamond substrates, generally varied in the range of 5–25 cm^−1^. The smaller the FWHM, the higher the diamond crystal quality [19,20]. It can be seen from Table 2 that the FWHM varied in the range of 7–9 cm^−1^, and the crystal quality was generally good. The B1 sample (R_a_ 0.03 μm, 16 sccm, 8 h, 3 mm) had the smallest FWHM; the C3 sample (R_a_ 0.4 μm, 16 sccm, 12 h, 4.5 mm) had the maximum FWHM. Through the analysis of FWHM and residual stress, it can be seen that the quality of sample set B’s diamond film was better.

#### 3.1.4. XRD Patterns of Diamond Films

**Figure 5:**
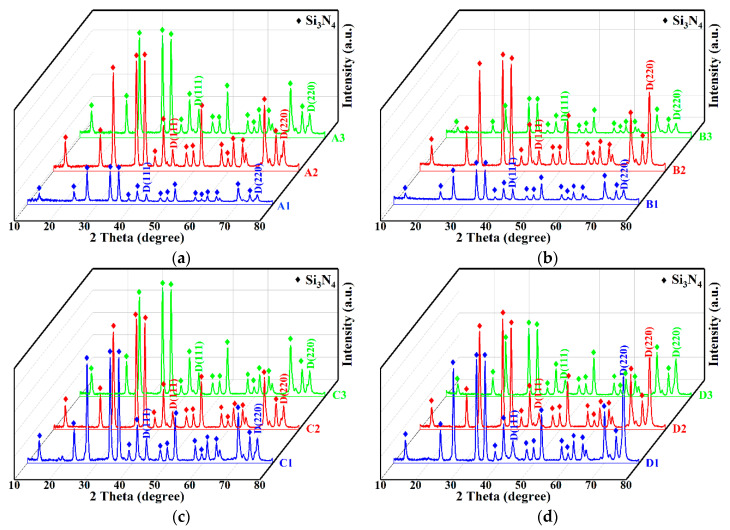
XRD patterns of diamond films with different roughnesses. 1-R_a_ 0.03 μm, 2-R_a_ 0.1 μm, 3-R_a_ 0.4 μm: (**a**) A1–A3 (16 sccm, 8 h, 4.5 mm); (**b**) B1–B3 (16 sccm, 8 h, 3 mm); (**c**) C1–C3 (16 sccm, 12 h, 4.5 mm); (**d**) D1–D3 (40 sccm, 5 h, 4.5 mm).

When the lattice constant of the HFCVD diamond film is close to that of natural diamond, it indicates that the quality of the diamond film is good, but the stress in the film causes lattice distortion, resulting in the shift of the lattice constant [21]. Figure 5 shows the XRD patterns of the diamond films with substrates of different roughnesses. According to the standard card (PDF06-675), the diffraction peaks at 2θ = 43.9° and 75.3° represent diamond (111) and crystal (220) planes [22], and other peaks can be assigned to the substrate material (Si_3_N_4_) because diamond films are relatively thin and easily penetrated by X-ray [23]. It can be seen from Figure 5 that all the diamond films had (111) and (220) characteristic peaks. When the CH_4_ concentration increased from 16 sccm to 40 sccm, it can be seen from Figure 5d that the intensity of the (220) diffraction peak increased significantly, indicating that the preferred orientation of the crystal plane of diamond changed from (111) to (220). It can also be seen from Figure 5d that, when the CH_4_ concentration was 40 sccm, the width at half the maximum of the (111) crystal plane became larger, which is due to the increase in the CH_4_ concentration, the increase in the secondary nucleation density, and the smaller diamond grains; the grain defect density can also cause this change. In Figure 1, the transformation of the diamond crystal plane from (111) to (220) and the decrease in grain size can also be seen. Figure 5a–c shows the XRD patterns of diamond thin films obtained by changing the deposition time and distance on substrates with different roughnesses. It can be seen that the patterns were relatively similar, the influence was small, and the CH_4_ concentration was the most influential factor.

### 3.2. Friction and Wear Properties of Diamond Films

**Figure 6:**
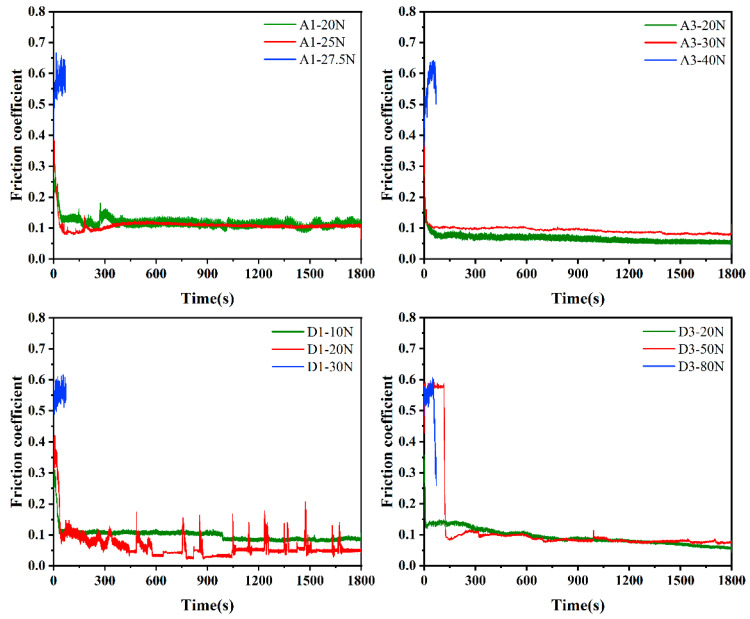
Friction coefficients of MCD and NCD with different loads and roughnesses.

**Figure 7:**
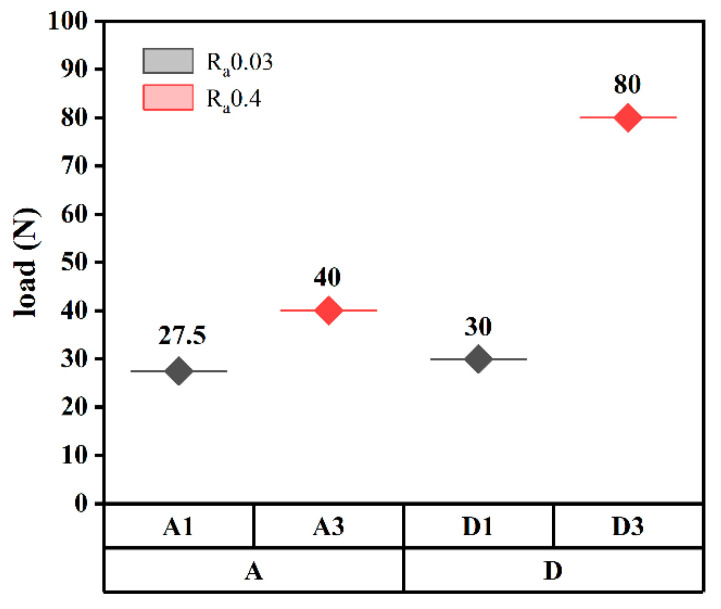
Diamond film separation load threshold.

Figure 6 shows the friction coefficients of the MCD and NCD of the substrates with different roughnesses under different loads. A1 and A3 were MCD with a substrate roughness of 0.03 μm, and D1 and D3 were NCD with a substrate roughness of 0.4 μm. A normal load from small to large was applied to the diamond film, and the dry friction and wear tests were carried out for 30 min. If the film delamination was observed, the test was ended [24,25]. As shown in Figure 7, the normal loads of film separation of A1, A3, D1, and D3 were 27.5 N, 40 N, 30 N, and 80 N, respectively. As can be seen from Figure 6, before the film was delaminated, that is, when the normal load was relatively small, the friction coefficient was relatively small: around 0.1. When the film came off and delaminated, the friction coefficient was relatively large: around 0.6. Combined with Figure 7, it can be seen that the delamination loads of A3 and D3 films were larger than those of A1 and D1, indicating that the adhesion of diamond films deposited on the R_a_ 0.4 μm substrate was higher. The delamination load of the D1 film was greater than that of A1, and the delamination load of D3 was greater than that of A3, indicating that the adhesion of NCD was greater than that of MCD. The delamination load of D3 was 80 N, which was much larger than that of A3, indicating that the adhesion of NCD deposited on the R_a_ 0.4 μm substrate with higher roughness was stronger.

**Figure 8:**
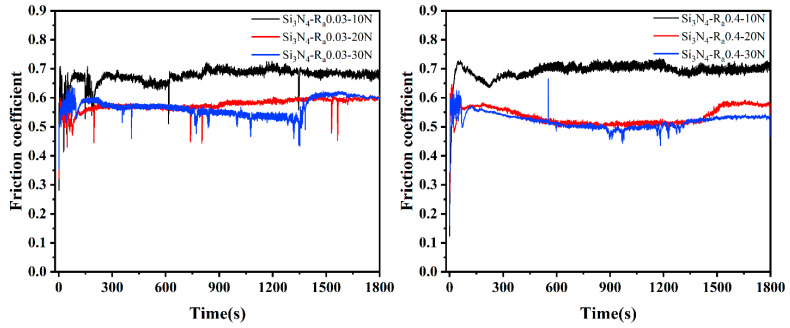
Friction coefficient of Si_3_N_4_ substrate with different roughnesses.

Figure 8 shows the friction coefficient curves of uncoated Si_3_N_4_ substrates with different roughnesses under different loads. It can be seen from the figure that the friction coefficients of diamond films deposited on substrates with roughnesses of 0.03 μm and 0.4 μm were around 0.5–0.7, which is relatively large, which also provides a basis for judging film delamination in Figure 6. It can be seen from Figure 6 that the friction coefficient of the film without separation was about 0.1, which is much smaller than the 0.5–0.7 of the Si_3_N_4_ substrate, indicating that the deposition of MCD and NCD on the Si_3_N_4_ substrate can reduce the friction coefficient and improve the friction performance.

**Figure 9:**
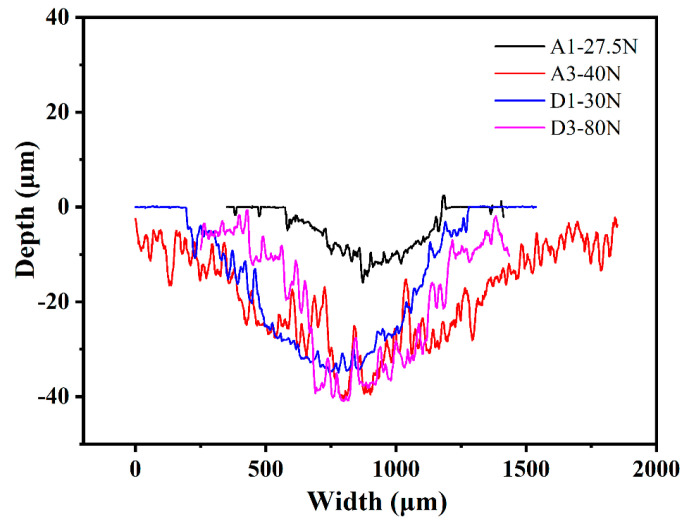
Cross-sectional profiles of the wear track of diamond films.

The wear scar profile of the worn surface of the film was measured by a white-light interferometer, and the wear rate of the worn film was calculated. Figure 9 shows the scratch trajectories formed under the delamination load of the MCD and NCD films with substrates of different roughnesses. The wear rate of the diamond film was calculated by Formula 2 [26]:

(2) $$K=\frac{V}{LF}$$

*K* is the wear rate of the film, *V* is the wear volume of the film, and the unit is mm^3^; *L* is the total sliding stroke of the wear experiment, and the unit is m; *F* is the normal load applied to the film, and the unit is N.

The wear rates of the A1, A3, D1, and D3 diamond films were 4.68 × 10^−4^ mm^3^/mN, 1.05 × 10^−3^ mm^3^/mN, 5.34 × 10^−4^ mm^3^/mN, and 5.44 × 10^−4^ mm^3^/mN, respectively. A1 was MCD with a substrate roughness of 0.03 μm, which has good crystal quality and a low wear rate. Combined with Figure 9, it can also be seen that the width and depth of the wear mark curve of A1 were lower, indicating that it has good wear resistance. The wear rates of MCD and NCD with a substrate roughness of 0.03 μm were lower, indicating that there is a certain relationship between the wear rate and substrate roughness.

**Figure 10:**
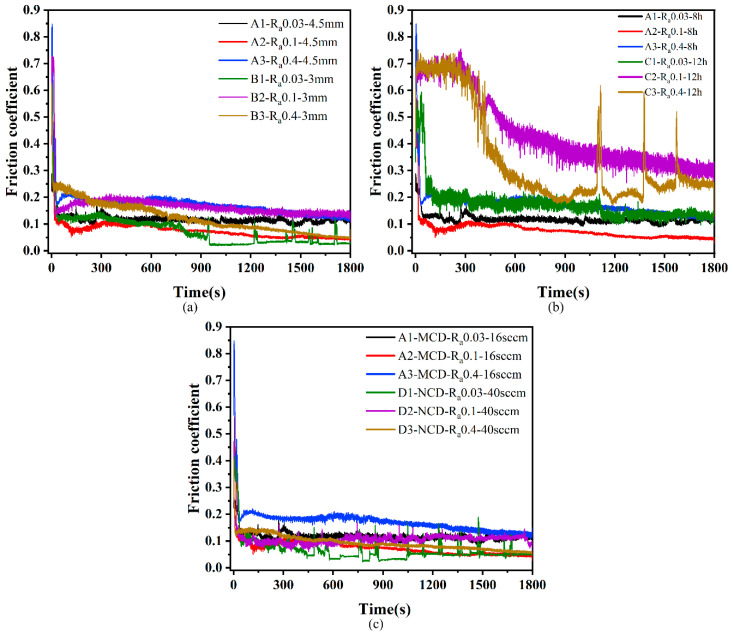
Friction coefficient curves of diamond films with substrates of different roughnesses under 20 N load: (**a**) friction coefficient curves of diamond films at different deposition distances; (**b**) friction coefficient curves of diamond films at different deposition times; (**c**) friction coefficient curves of MCD and NCD.

**Figure 11:**
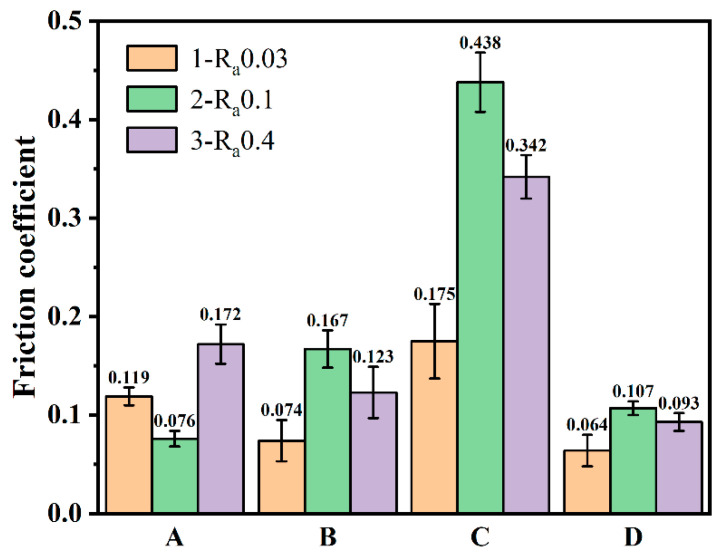
Bar diagram of friction coefficient of diamond films.

**Figure 12:**
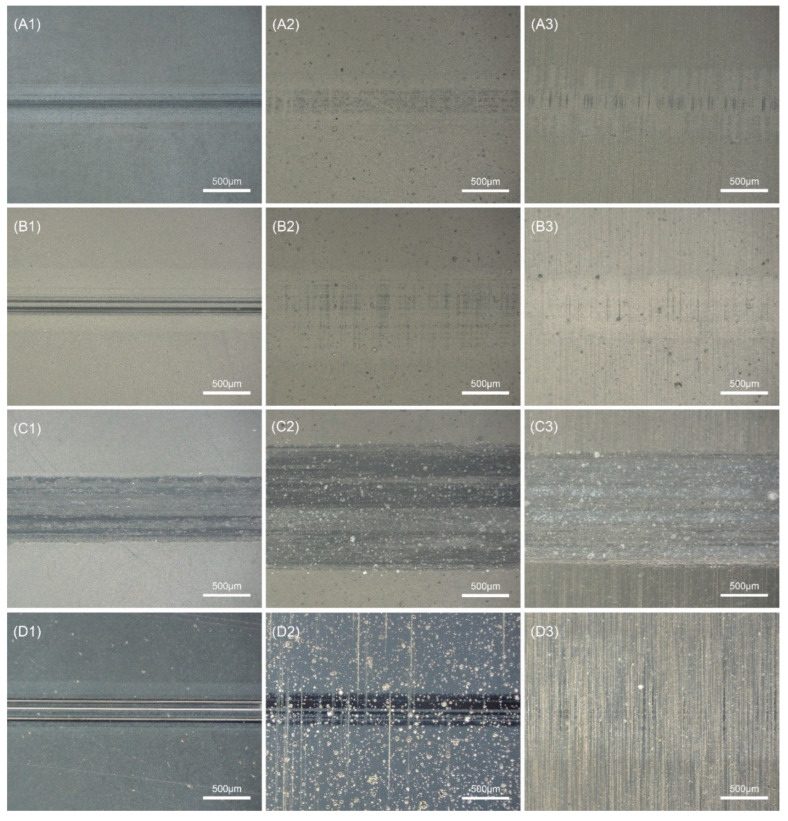
Wear tracks of diamond films.

Figure 10 shows the coefficient of friction curves under dry friction conditions with a 20 N load applied to the prepared diamond films. It can be seen that the variation trend of the friction coefficient of all samples was the same, which was characterized by a relatively strong and narrow peak at the beginning, mainly due to the relatively low hardness of the Si_3_N_4_ ceramic ball surface during the friction sliding process. There was a plowing action of sharp diamond grain peaks, and, after a period of friction, the subsurface formed a transfer film on the diamond crystals [27,28]. After the running-in period, the stable wear stage was entered, and the friction coefficient also became stable. Figure 11 shows the average friction coefficient of the prepared diamond film after the friction and wear test, which reflects the friction properties of the diamond film under different deposition conditions. Figure 12 shows the OM images of wear tracks.

Figure 10a shows the friction coefficient curves of diamond films deposited at different deposition distances. It can be seen that the friction coefficient curves for deposition distances of 3 mm and 4.5 mm were roughly the same. It can be seen from Figure 11 that the average friction coefficients of A and B were very similar, ranging from 0.07 to 0.17. Among them, the friction coefficient of B1 presented a sawtooth shape in the later stage of entering the stable stage. This may be because the micron columnar grains interacted with the counterbody to cause the diamond grains to fall off. After falling off, the grains fall on the opposite grinding surface between the diamond film and the Si_3_N_4_ ceramic ball. During the reciprocating sliding process of the ceramic ball, the friction coefficient fluctuates when it encounters the diamond grains. Combining sets A and B in Figure 12, it can be seen that the wear track morphology was also very similar, and there was no overall shedding of the diamond coating. The results show that the deposition distance has little effect on the friction coefficient.

Figure 10b shows the friction coefficient curves of diamond films deposited at different deposition times. Among them, C2 and C3 with a deposition time of 12 h did not enter a stable state until about 800 s, but the friction coefficient of the stable state was still larger than that of A1–A3 with a deposition time of 8 h. Based on Figure 8, it can be seen that the average friction coefficient of set C was larger than that of set B, and the average friction coefficient of B2 and C1 was close, which may be because the roughness of the C1 substrate was relatively low. It can be seen from the C set of Figure 12 that the C1–C3 diamond films were peeled off. This shows that the adhesion of the diamond film with a deposition time of 12 h was less than that of the diamond film with a deposition time of 8 h. The increase in the friction coefficient may be due to the direct grinding of the Si_3_N_4_ ball with the Si_3_N_4_ substrate after the delamination of the diamond film. Due to the existence of the transfer film, the friction coefficient of the C set was lower than the friction coefficient (0.5–0.7) of the friction coefficient between the Si_3_N_4_ ball and the Si_3_N_4_. The results show that, within a certain deposition range, too long a deposition time increases the friction coefficient and reduces the bonding force between the film and the substrate.

Figure 10c shows the friction coefficient curves of MCD and NCD. The friction coefficients of the two were similar. It can be seen that the friction coefficient of A3 was the highest after stabilization, and the friction coefficient of D1 was the lowest. However, it experienced a sudden change in the friction coefficient in the stable stage, and the reason is similar to that of B1 in Figure 10a. This is because the nano-diamond grains fall off under the action of mechanical force when the nano-diamond grains are ground against the Si_3_N_4_ ball. The Si_3_N_4_ ball encounters the diamond grains during the reciprocating sliding process, which causes a sudden, short-term change in the friction coefficient. It can be seen from sets A and D in Figure 11 that the average friction coefficient of NCD as a whole was smaller than that of MCD. This is because the grain size in NCD decreases, the crystal quality deteriorates, the friction area increases, the nano-diamond grains are smoothed, and some of the grains fall off. The chips and the falling grains form a transfer film at the friction interface, and, because of its smooth surface, low roughness, and the lubricant of the amorphous carbon phase distributed at the diamond grain boundary, its friction coefficient is lower than MCD.

By analyzing the overall average friction coefficient in Figure 11, it can be seen that the friction coefficient of NCD was lower than that of MCD deposited under different conditions. This also confirms the conclusion drawn from Figure 10c. From the perspective of substrates of different roughnesses, it can be concluded that the relationship between the average friction coefficient of the diamond film is COF_Ra0.03μm_ < COF_Ra0.4μm_ < COF_Ra0.1μm_. This also shows that the tribological properties of the diamond film are not proportional to the roughness of the substrate. The average friction coefficient is the smallest when the roughness of the Si_3_N_4_ substrate is 0.03 μm, and the average friction coefficient is the largest when the roughness of the Si_3_N_4_ substrate is 0.1 μm.

Figure 12 indicates that sets A, B, and D had slight scratches, which did not damage the surface of the diamond film. The C1–C3 diamond films were all peeled off, which is in full agreement with the average friction coefficient in Figure 10. The wear scar width of C2 was larger than C1 and C3, which is also related to the average friction coefficient. It can also be seen from Figure 12 that the one (R_a_ 0.03 μm) scratch in each set was the narrowest, corresponding to its smaller average friction coefficient.

## 4. Conclusions

This paper reports the tribological properties of MCD and NCD diamond films deposited on Si_3_N_4_ substrates with different roughnesses using the HFCVD technique.

The surface morphologies and cross-sectional morphologies of the diamond films were obtained by SEM. MCD diamond deposited at a 16 sccm CH_4_ concentration has larger grains and better crystal quality. The NCD diamond grains deposited under 40 sccm CH_4_ concentration are finer, and the grain size is tens of nanometers. The growth model was changed from a columnar growth to the nano-grain accumulation growth mode, and the growth rate was greatly improved. The grain size increased (2–3 μm) when the deposition distance was closer (3 mm). Changes in the deposition time had little effect on the surface topography. By analyzing the roughness of the diamond film, it was found that the growth of the film matched its starting surface.

Raman spectra showed that the residual stress of diamond film deposited on the R_a_ 0.4 μm substrate was smaller, and the minimum residual stress was −0.02 GPa. Among them, the FWHM of the diamond characteristic peaks was small, in the range of 7–9 cm^−1^, indicating that the crystalline quality of the diamond film is good. The XRD results showed that the preferred orientation of the diamond film changes from (111) to (220) when the CH_4_ concentration increases from 16 sccm to 40 sccm.

The tribological properties of diamond films were studied by reciprocating friction and wear tests. The adhesion of MCD and NCD deposited on the R_a_ 0.4 μm substrate was greater when studying the load delamination threshold of diamond films. The lowest wear rates of diamond films with a substrate roughness of 0.03 μm were 4.68 × 10^−4^ mm^3^/mN and 5.34 × 10^−4^ mm^3^/mN. When the film was deposited for a long time (12 h), its friction coefficient increased, and the adhesion between the film and the substrate was reduced. The diamond film deposited on the Si_3_N_4_ substrate reduced the friction coefficient of the material and improved the friction performance. The average friction coefficient of NCD (0.064, 0.107, and 0.093) was lower than that of MCD, which depended on the grain size, crystalline quality, and roughness. The diamond film deposited on the substrate with a roughness of 0.03 μm had excellent mechanical and tribological properties, such as small residual stress, a low wear rate, and a low average friction coefficient.

## Figures and Tables

**Table 1:** Deposition parameters.

Sample Number	Roughness (μm)	CH_4_ Concentration (%)	Deposition Time (h)	Deposition Distance (mm)	Gas Flow (sccm)	
					CH_4_	H_2_
A1	0.03	2	8	4.5	16	800
A2	0.1	2	8	4.5	16	800
A3	0.4	2	8	4.5	16	800
B1	0.03	2	8	3	16	800
B2	0.1	2	8	3	16	800
B3	0.4	2	8	3	16	800
C1	0.03	2	12	4.5	16	800
C2	0.1	2	12	4.5	16	800
C3	0.4	2	12	4.5	16	800
D1	0.03	5	5	4.5	40	800
D2	0.1	5	5	4.5	40	800
D3	0.4	5	5	4.5	40	800

**Table 2:** Results of the Raman spectra of diamond films.

Sample Number	A1	A3	B1	B3	C1	C3	D1	D3
FWHM/cm^−1^	9.08	8.86	7.91	8.36	8.64	9.55	9.01	9.37
*v*/cm^−1^	1330.98	1331.24	1332.29	1332.03	1330.58	1330.69	1332.75	1332.54
*σ*/GPa	+0.58	+0.43	−0.16	−0.02	+0.80	+0.74	−0.42	−0.30

## Data Availability

Not applicable.
